# Editorial

**Published:** 1872-09-01

**Authors:** 


MEDICAL EXAMINER.

*A Semi-Monthly Journal of Medical Sciences.*

EDITED BY

N. S. DAVIS, M. D., AND F. H. DAVIS, M. D.

Chicago, September 1st, 1872.

### EDITORIAL.

CHICAGO MEDICAL COLLEGE.—The commencement of the next regular annual course of instruction in this college, will be on Tuesday evening, October 1st, 1872. The Introductory Lecture will be given by Prof. D. T. Nelson. We wish our readers to be particular to note the day, because, formerly, the first Monday in October has been the time for commencing the term, and from an oversight it had been allowed to remain so stated in the standing advertisement of the college, in our advertising columns.

CUNDURANGO AND CANCER.—It will be remembered that among the recommendations of this remedy, heralded over the country on its first introduction to the notice of the profession and the community, was a certificate of its having cured the mother of Vice-President Colfax. The genuineness of the cure can be inferred from the following recent telegraphic announcement:

"SOUTH BEND, Ind., Aug. 11.

"Mrs. Matthews, mother of Vice-President Colfax, died this afternoon of cancer."

DR. PARVIN.—We notice that Prof. Theophilus Parvin has resigned his position in the University of Louisville. Dr. Parvin is one of our ablest and most reliable medical teachers.

OINTMENT FOR PILES.—M. F. Guym, of the Necker Hospital, Paris, prescribes in painful hæmorrhoids an ointment compound of one part of extract of belladonna, two of extract of rhatamy, and fifteen of lard.—*The Doctor*.

